# Supplementary figures and images for: Leucine alleviates cytokine storm syndrome by regulating macrophage polarization via the mTORC1/LXRα signaling pathway
Source: eLife. 2024 Mar 5;12:RP89750. doi: 10.7554/eLife.89750 (PMC10942637; doi:10.7554/eLife.89750)

Figure 3F

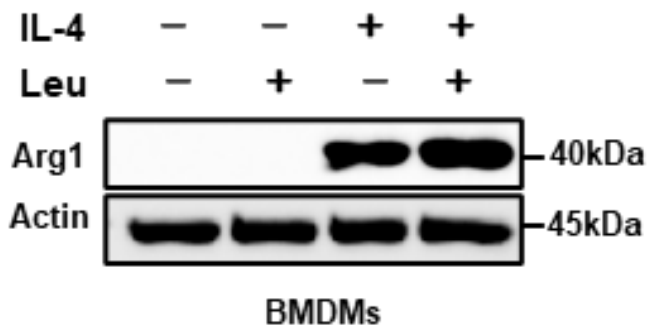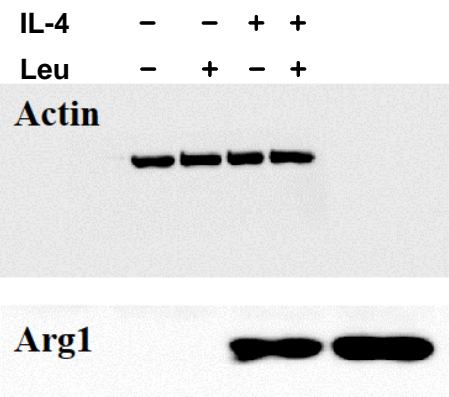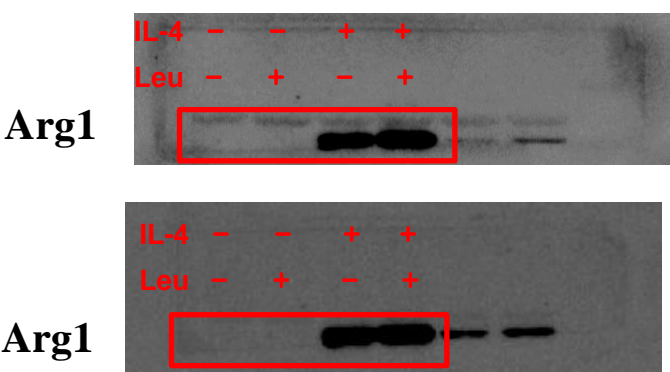

Supplement: Figure 3—source data 4. [file elife-89750-fig3-data4.zip › Figure 3-source data 4.pdf]

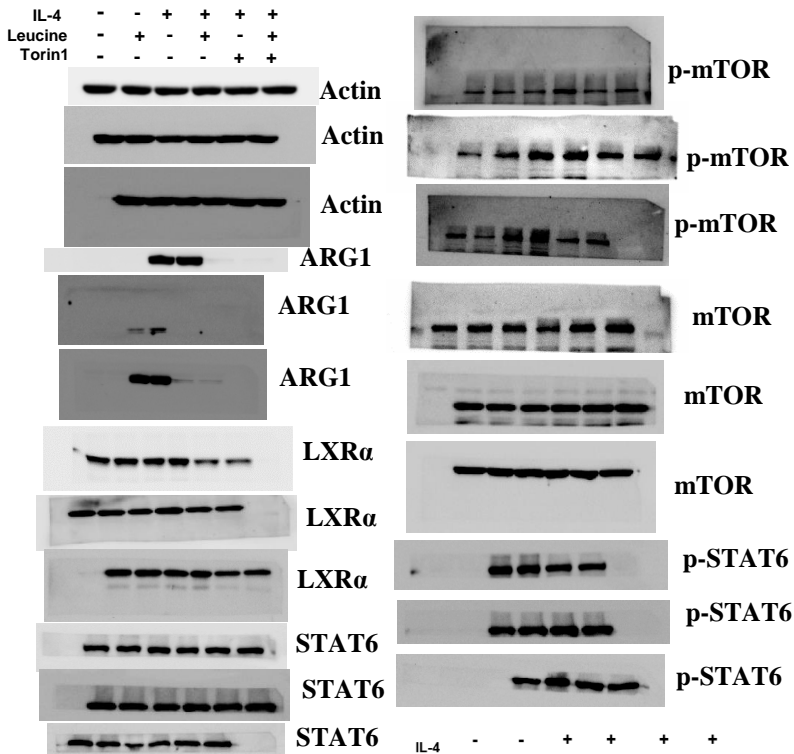

**Figure 4A**

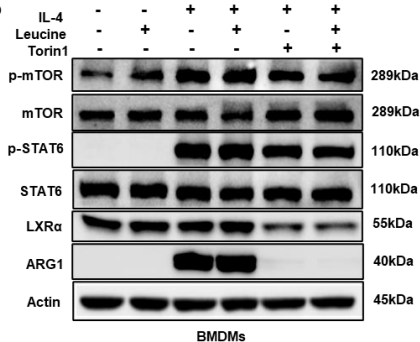

Supplement: Figure 4—source data 1. [file elife-89750-fig4-data1.zip › Figure 4-source data 1.pdf]

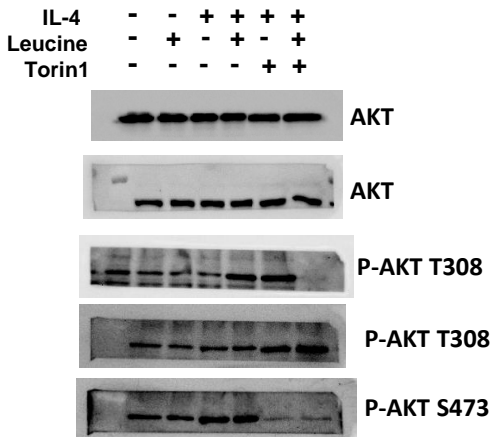

**Figure 4-supplementary figure 2C**

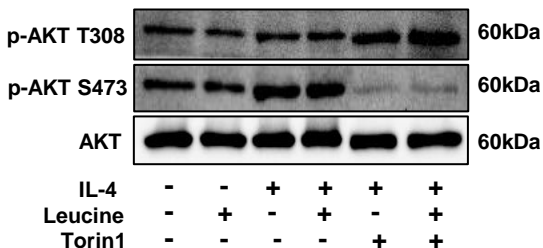

Supplement: Figure 4—figure supplement 1—source data 3. [file elife-89750-fig4-figsupp1-data3.zip › Figure 4-figure supplement 2-source data 3.pdf]

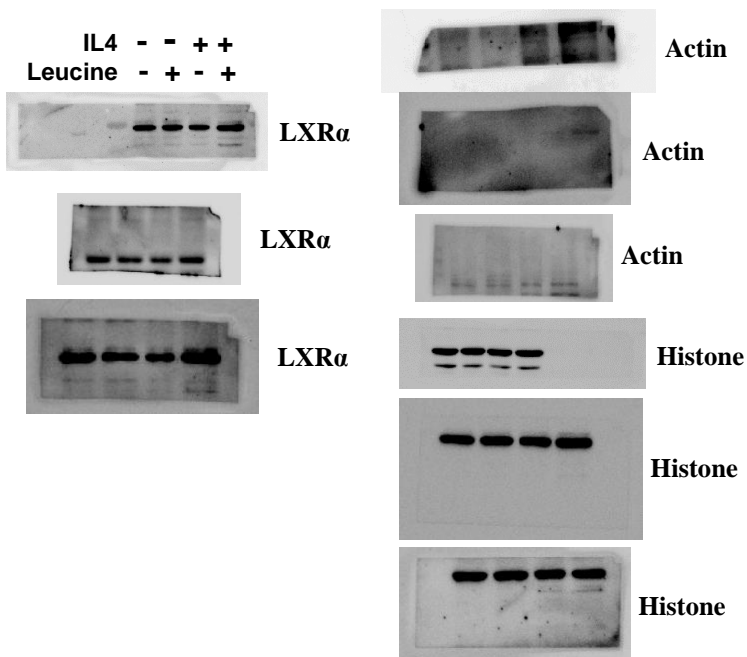

**Figure 5B**

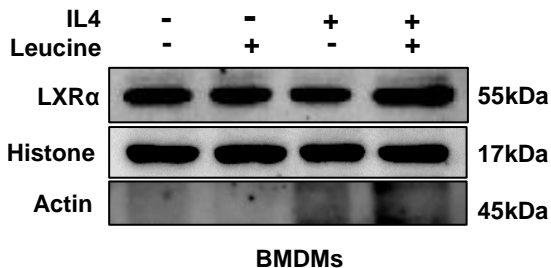

Supplement: Figure 5—source data 2. [file elife-89750-fig5-data2.zip › Figure 5-source data 2.pdf]
