# Supplementary material for: Leucine alleviates cytokine storm syndrome by regulating macrophage polarization via the mTORC1/LXRα signaling pathway
Source: eLife. 2024 Mar 5;12:RP89750. doi: 10.7554/eLife.89750 (PMC10942637; doi:10.7554/eLife.89750)
Supplement: Figure 5—source data 1. [file elife-89750-fig5-data1.zip › Figure 5-source data 1.pdf]

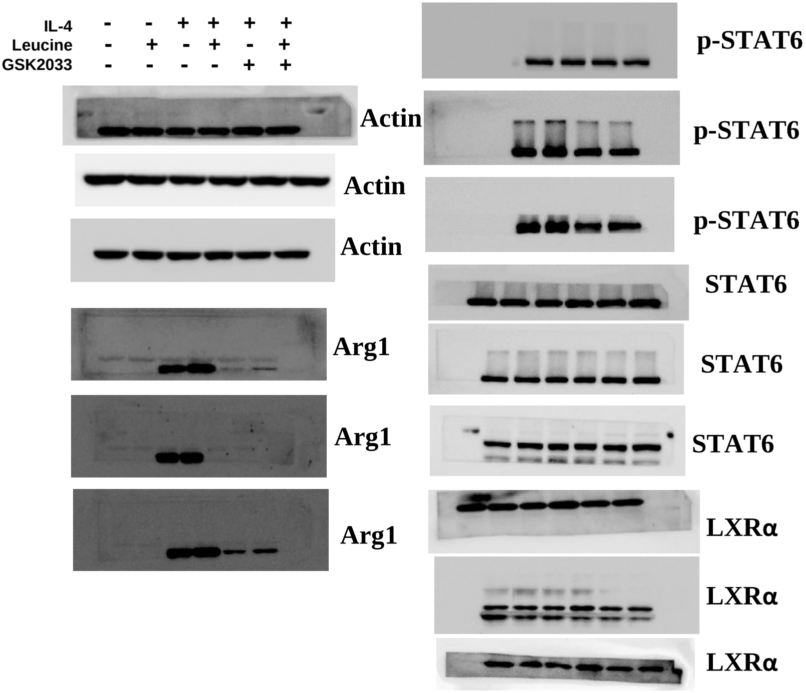
IL-4 - - + + + +
Leucine - + - + - +
GSK2033 - - - - + +
Actin
Actin
Actin
Arg1
Arg1
Arg1
p-STAT6
p-STAT6
p-STAT6
STAT6
STAT6
STAT6
LXRα
LXRα
LXRα

Figure 5A

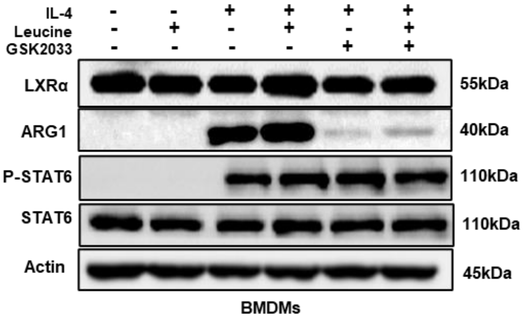
IL-4 - - + + + +
Leucine - + - + - +
GSK2033 - - - - + +
LXRα
55kDa
ARG1
40kDa
P-STAT6
110kDa
STAT6
110kDa
Actin
45kDa
BMDMs
