# Supplementary material for: Leucine alleviates cytokine storm syndrome by regulating macrophage polarization via the mTORC1/LXRα signaling pathway
Source: eLife. 2024 Mar 5;12:RP89750. doi: 10.7554/eLife.89750 (PMC10942637; doi:10.7554/eLife.89750)
Supplement: Supplementary file 1. [file elife-89750-supp1.docx]

**Supplementary File 1.** qPCR primer sequences.

| **Gene** | **Forward 5′-3′** | **Reverse 5’-3’** |
| --- | --- | --- |
| *Arg1* | AGCACTGAGGAAAGCTGGTC | CAGACCGTGGGTTCTTCACA |
| *Mgl1* | TGCAACAGCTGAGGAAGGACTTGA | AACCAATAGCAGCTGCCTTCATGC |
| *Mgl2* | GCATGAAGGCAGCTGCTATTGGTT | TAGGCCCATCCAGCTAAGCACATT |
| *Fizz1* | TCCAGCTGATGGTCCCAGTGAATA | ACAAGCACACCCAGTAGCAGTCAT |
| *Ym1* | AGAAGGGAGTTTCAAACCT | GTCTTGCTCATGTGTGTAAGTGA |
| *Il6* | AAAATTTCCTCTGGTCTTCTGGAGT | TTCTGTGACTCCAGCTTATCTCTTG |
| *Il1β* | GCTTCCTTGTGCAAGTGTCTGA | TCAAAAGGTGGCATTTCACAGT |
| *Mcp1* | CCACTCACCTGCTGCTACTCA | TGGTGATCCTCTTGTAGCTCTCC |
| *Inos* | CAGGTCTTTGACGCTCGGAA | GCCTGAAGTCATGTTTGCCG |
| *Nlrp3* | ATTACCCGCCCGAGAAAGG | TCGCAGCAAAGATCCACACAG |
| *Tnfα* | TCTCATGCACCACCATCAAGGACT | ACCACTCTCCCTTTGCAGAACTCA |
| *Ppia* | TGCCCGCAAGTCAAAAGAAAT | ACTGAATGGCTGGATGGCAAG |
