# Supplementary material for: Leucine alleviates cytokine storm syndrome by regulating macrophage polarization via the mTORC1/LXRα signaling pathway
Source: eLife. 2024 Mar 5;12:RP89750. doi: 10.7554/eLife.89750 (PMC10942637; doi:10.7554/eLife.89750)
Supplement: Supplementary file 2. [file elife-89750-supp2.docx]

**Supplementary File 2.** Antibody information.

| **Antibodies** | **Source** | **Identifier** |
| --- | --- | --- |
| Purified Rat Anti-Mouse CD16/CD32 | BD Pharmingen™ | Cat # 553141 |
| FITC Rat Anti-Mouse CD45 | BD Pharmingen™ | Cat # 553080 |
| Alexa Fluor® 647 Rat Anti-Mouse F4/80 | BD Pharmingen™ | Cat # 565853 |
| PerCP-Cy™5.5 Rat Anti-CD11b (M1/70) | BD Pharmingen™ | Cat # 550993 |
| Alexa Fluor®647 Rat Anti-Mouse CD206 | BD Pharmingen™ | Cat # 565250 |
| APC/Cyanine7 anti-mouse CD86 | Biolegend | Cat # 105030 |
| β-Actin Rabbit mAb | CST | Cat # 4970S |
| LXR alpha Antibody | NOVUS | Cat # NBP2-66938 |
| Arginase-1 XP® Rabbit mAb | CST | Cat # 93668 |
| Stat6 Rabbit mAb | CST | Cat # 5397S |
| Phospho-Stat6 (Tyr641) Rabbit mAb | CST | Cat # 56554S |
| 4E-BP1 Rabbit mAb | CST | Cat # 9644S |
| Phospho-4E-BP1 Rabbit mAb | CST | Cat # 13443S |
| Akt (pan) (11E7) Rabbit mAb | CST | Cat # 4691S |
| Phospho-Akt (Ser473) XP® Rabbit mAb | CST | Cat # 4060S |
| Phospho-Akt (Thr308) XP® Rabbit mAb | CST | Cat # 13038S |
| p70 S6 Kinase Rabbit mAb | CST | Cat # 2708S |
| Phospho-Drosophila p70 S6 Kinase (Thr398) Antibody | CST | Cat # 9209S |
